# An approach to Repeat Breeder Syndrome. Cervical mucus immunoglobulin content does not alter sperm motility

**DOI:** 10.1590/1984-3143-AR2024-0105

**Published:** 2025-04-14

**Authors:** Sofía Lara Villar, Uxía Yáñez, Jacobo Álvarez, Mar Alvariño, Carlos Carmelo Pérez-Marín, Juan José Becerra, Pedro García Herradón, Ana Isabel Peña, Luis Ángel Quintela

**Affiliations:** 1 Unidade de Reprodución e Obstetricia, Departamento de Patoloxía Animal, Facultade de Medicina Veterinaria, Universidade de Santiago de Compostela, Lugo, España; 2 Departmento de Medicina y Cirugía Animal, Facultad de Medicina Veterinaria, Universidad de Córdoba, Córdoba, España; 3 Instituto de Biodiversidade Agraria e Desenvolvemento Rural, Universidade de Santiago de Compostela, Lugo, España

**Keywords:** repeat breeder cow, dairy cattle, cervical mucus, spermatozoa, immunity

## Abstract

Repeat breeder (RB) syndrome plays a detrimental role on fertility and economic performance of dairy farms. Regarding its multifactorial origin, it has been stated that an immunological response in the female genital tract may impair sperm viability. Therefore, the aim of this study was to evaluate the concentration of immunoglobulins in cervical mucus of RB cows and its influence on sperm motility. Fifteen fertile cows and 32 RB cows were included in the study. Cervical mucus samples were collected at the time of artificial insemination (AI). The concentration of IgG and IgA was determined by radial immunodiffusion. Sperm motility in cervical mucus was evaluated by CASA system. Our results showed no significant differences between cows with or without RB syndrome regarding the concentration of immunoglobulins in cervical mucus and sperm motility. The only factor affecting sperm motility was time. Consequently, it may be probable that a local immune response against spermatozoa is not one common cause of RB syndrome.

## Introduction

Repeat breeder (RB) syndrome is defined as the lack of conception, after three or more artificial inseminations (AI), in absence of appreciable genital tract disease. It is considered a main reproductive problem affecting fertility and the economic performance of dairy farms (Nowicki, 2021). The cause of this phenomenon is multifactorial, including nutritional issues, hormonal alterations, and immunological mechanisms (Abraham, 2017; Perez-Marin et al., 2012). In this regard, spermatozoa are foreign cells to the female organism and may induce an immune response that impairs fertilisation. In humans, it has been observed that isoimmunization (immune response against spermatozoa) plays a main role in more than 30% of infertility cases (Brazdova et al., 2014). However, this statement is not clear for animal reproduction.

To avoid isoimmunization, there are several body fluids capable of suppressing this response, such as seminal plasma, follicular and uterine fluid, and cervical mucus (Landers et al., 1994). Focusing on cervical mucus, it should be considered that its composition, as well as its physical and biochemical characteristics, are affected by the oestrus cycle and health status of the female (López-Gatius et al., 1993), and may be key in the success or failure of pregnancy (Siregar et al., 2019). In this regard, it has been stated that high levels of anti-sperm antibodies in cervical mucus affect sperm-mucus interactions, with a reduction in sperm motility (Jarora et al., 2016; Lazarevic et al., 2013).

Although in humans it has been proved that sperm-mucus interactions can be altered by immunoglobulins localised in the cervical mucus (Nakano et al., 2015), in veterinary medicine inflammation is not considered a main reason for subfertility of cows at AI, and these investigations are scarce. Consequently, the aim of this study was to evaluate the concentration of immunoglobulins in cervical mucus of RB cows and its influence on sperm motility.

## Methods

### Animals

Forty-seven Holstein cows were included in the study: 15 healthy cows (pregnant after the first or second AI) and 32 RB cows (animals that have calved at least once, without appreciable alterations on their genital tract, and that needed 3 or more AI to become pregnant). All animals belonged to the same farm, with 380 cows in milk. Mean parity was 2.19 (1.73 for healthy cows and 2.4 for RB cows).

Samples were collected from March to July 2019, on Thursday and Friday, when AI was performed following the fixed-timed artificial insemination Ovsynch protocol. After sample collection, cows were monitored using on-farm reproductive software, placing the cows in the corresponding group according to the number of AI needed to become pregnant (Healthy: <3; RB: ≥3). After pregnancy diagnosis, cows were classified as Pregnant (n = 14) and Non-pregnant (n = 33).

### Sample collection

**Figure 1:**
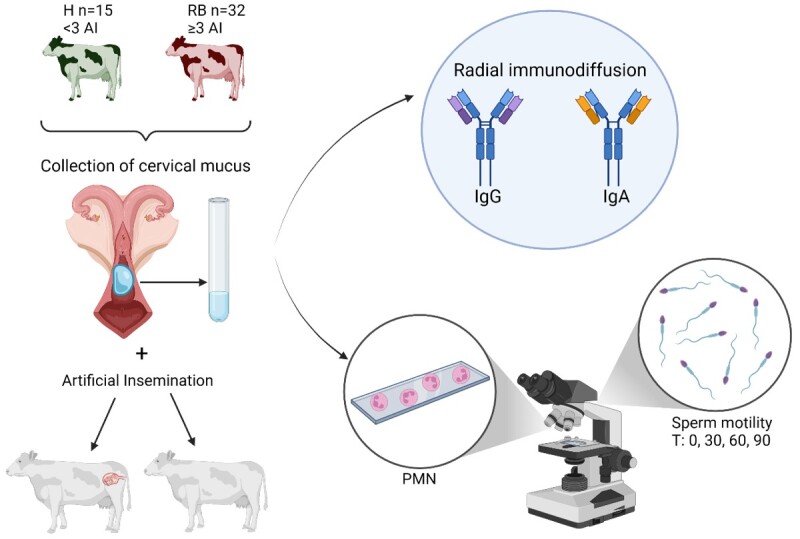
Diagram of the study design, representing the animals included (H: healthy, RB: repeat breeder), sample collection, sample analysis (determination of the concentration of IgG, IgA, and polymorphonuclear neutrophils (PMN) in cervical mucus and effect on sperm motility at different times (T)), and outcome (pregnant/non-pregnant) of the artificial insemination (AI).

The experiment was exempted from ethics review, as stated by the Ethics Committee of the Rof Codina Veterinary Teaching Hospital, as this practice fell into the exceptions referred in Article 2 (5.f) of the national decree-law RD53/2013 (2010/63/EU Directive). Cervical mucus samples were collected just before the AI (Figure 1). First, the perineum and vulva were cleaned with dry paper. Next, a gloved hand was introduced in the vagina, the cervical mucus was collected and placed in a 50 mL tube and transported in refrigeration to the laboratory in < 1 hour. Then, it was grounded with a Polytron (PT2000, Kinematica, Malters, Switzerland) and diluted 1:2 with physiological saline solution. Finally, it was frozen in aliquots until analysis. Regarding the semen samples used, they were bought in a local commercial distributor (Xenética Fontao, Lugo, Spain).

### Sample analysis

The concentration of IgG and IgA in cervical mucus was determined by radial immunodiffusion essay (Bovine IgG Test Kit and Bovine IgA Test Kit, Eurovet Veterinaria S.L., Madrid, Spain), following the manufacturer’s instructions. Cows were divided into two groups according to the cervical mucus concentration (mg/dL) of IgG: <180 (n=45) and >180 (n=2), according to the concentration of the lowest control solution supplied by the manufacturer (180 mg/dL); similarly, for IgA concentration, cows were classified as <53 (n=27) and >53 (n=10), according to the concentration of the lowest control solution supplied by the manufacturer (53 mg/dL). It should be noted that it was not possible to obtain information about IgA concentration for 10 cows.

In addition, the percentage of polymorphonuclear neutrophils (%PMN) was also determined for each cow. A drop of cervical mucus was smeared on a slide and stained with Diff Quick (Quick Panoptic kit, Quimica Clinica Aplicada S.A., Tarragona, Spain). Thereafter, the slide was observed under an optical microscope (CHT, Olympus Iberia S.A.U., Barcelona, Spain) at 100x magnification and a total of 150-200 nucleated cells were counted to calculate the %PMN. The %PMN was considered either as continuous and categorical variable (absence (0) or presence (1) of PMN).

The sperm motility evaluation was performed by CASA system (Sperm Class Analyzer 6.1.0; Microptic, Barcelona, Spain). First, the cervical mucus samples were homogenized using a mechanical agitator, and semen samples were thawed in a water bath at 37ºC for 30 s. Next, 90 µL of cervical mucus and 10 µL of semen were placed in a 1.5 mL reaction tube. In addition, a control trial for each semen sample (75 µL of saline solution and 25 µL of semen) was performed. A total of 5 µL of the reaction tube were placed on a Makler chamber (Israel Electrooptical Industry, Rehovot, Israel), and five microscopic fields were analysed per sample using a microscope (Eclipse E200, Nikon, Tokyo, Japan) supplied with a prewarmed stage at 37ºC and a negative phase-contrast objective at a magnification of 100x. This procedure was performed at 0, 30, 60 and 90 minutes after thawing. Objects incorrectly identified as spermatozoa were minimized on the monitor by using the playback function. Spermatozoa were classified into motile, progressive, rapid, and medium.

### Statistical analysis

Data were analysed using Pearson’s χ^2^ and General Linear Model (GLM) repeated measures tests. To perform the Pearson’s χ^2^ test, pregnancy and RB syndrome were chosen as dependent variables, and the concentration of IgG and IgA and PMN as independent variables. For the GLM test, the sperm motility categories were used as dependent variables, and pregnancy, RB syndrome, PMN, and time were included as factors.

All analyses were conducted in IBM SPSS Statistics version 28.0 for Windows (IBM Corp., Armonk, NY, USA). Differences were considered significant at p ≤ 0.05.

## Result

The results for Pearson’s χ^2^ analysis are displayed in Table 1. Regarding RB syndrome, most cows showed concentrations < 180 mg/dL of IgG and < 53 mg/dL of IgA in both healthy (93.3% and 71.4%) and RB (96.9% and 73.9%) groups, respectively (p > 0.05). Moreover, the %PMN was 29.6±41.7 for the healthy group and 58.9±34.9 for RB group (p=0.015). Additionally, 84.4% of RB cows and 40% of the healthy cows presented PMN in their cervical mucus (p < 0.01). Concerning Non-pregnant and Pregnant groups, similar results were obtained (97.0% and 92.9% for IgG <180 mg/dL and 69.2% and 81.8% for IgA <53 mg/dL, respectively, p > 0.05). The %PMN in Pregnant group was 55.9±37.0, while for non-pregnant cows was 35.3±40.8 (p > 0.05). However, when considering PMN as categorical variable, significant differences were observed, as 78.8% non-pregnant and 50.0% pregnant animals presented PMN in the cervical mucus (p = 0.048). Of the 47 cows included in the study, 14 were pregnant after the AI on the day of data collection (11 healthy and 3 RB, Conception rate = 29.8%).

**Table 1:** Proportion of (%) healthy (H) and Repeat breeder cows (RB) and non-pregnant (NP) or pregnant (P) considering the concentration (mg/dL) of Immunoglobulin G (IgG) and Immunoglobulin A (IgA) and the presence (1) or absence (0) of polymorphonuclear neutrophils (PMN) in cervical mucus.

		**H**	**RB**	**NP**	**P**
IgG	<180	93.3	96.9	97.0	92.9
	>180	6.7	3.1	3.0	7.1
IgA	<53	71.4	73.9	69.2	81.8
	>53	28.6	26.1	30.8	18.2
PMN	0	60.0^**^	15.6**	21.2^*^	50.0*
	1	40.0^**^	84.4^**^	78.8^*^	50.0^*^

* p<0.05; **p<0.01.

The results for GLM test are depicted in Table 2. The results for the control trial analysis were 58.98%, 56.16%, 52.96%, and 47.13% motile spermatozoa, 52.43%, 50.46%, 47.40%, and 51.53% progressive, 39.28%, 40.68%, 38.43%, and 34.63% rapid progressive, and 13.15%, 9.77%, 8.31%, and 6.90% medium progressive at times 0, 30, 60, and 90, respectively. The differences of mean progressive, rapid, medium, and motile spermatozoa were not statistically significant neither between healthy and RB cows, Non-pregnant and Pregnant groups, nor absence or presence of PMN (p > 0.05). However, significant differences (p < 0.001) were observed at different times.

**Table 2:** Proportion of mean spermatozoa (%) in healthy (H) and Repeat breeder cows (RB) and Non-pregnant (NP) and Pregnant (P) groups, measured at different times post-thawing (0, 30, 60, and 90 minutes).

	**Progressive**		**Rapid**		**Medium**		**Motile**	
**RBS**	**H(n=15)**	**RB(n=32)**	**H (n=15)**	**RB(n=32)**	**H(n=15)**	**RB(n=32)**	**H (n=15)**	**RB(n=32)**
Time 0	42.0±14.8^a^	43.2±15.8 ^a^	34.7±15.1 ^a^	33.6±12.7 ^a^	7.9±2.9 ^a^	9.6±4.3 ^a^	48.1±13.8 ^a^	49.2±15.9 ^a^
Time 30	28.8±10.0^b^	31.1±14.4 ^b^	23.2±10.1 ^b^	24.5±12.6 ^b^	5.7±2.0 ^b^	6.6±3.7 ^b^	33.7±8.8 ^b^	37.1±15.0 ^b^
Time 60	22.7±12.3^c^	21.1±14.3 ^c^	18.5±11.5 ^c^	15.5±11.3 ^c^	4.2±3.1 ^bc^	5.6±4.9 ^bc^	27.3±11.6 ^c^	26.6±16.8 ^c^
Time 90	19.2±12.1^d^	12.5±11.9 ^d^	15.4±10.6 ^d^	8.6±9.9 ^d^	3.8±2.7 ^c^	3.9±3.5 ^c^	23.1±12.7 ^d^	18.5±14.6 ^d^
**Pregnancy**	**NP(n=33)**	**P(n=14)**	**NP(n=33)**	**P(n=14)**	**NP(n=33)**	**P(n=14)**	**NP(n=33)**	**P(n=14)**
Time 0	42.4±15.8 ^a^	43.7±14.6 ^a^	33.1±12.9 ^a^	35.8±14.8 ^a^	9.3±4.5 ^a^	8.6±2.3 ^a^	48.8±15.9 ^a^	49.1±13.5 ^a^
Time 30	30.5±12.8 ^b^	30.1±14.3 ^b^	24.4±11.4 ^b^	23.4±13.0 ^b^	6.2±3.4 ^b^	6.7±3.1 ^b^	36.1±13.2 ^b^	35.9±14.1 ^b^
Time 60	21.9±13.6 ^c^	21.1±14.1 ^c^	16.2±10.9 ^c^	17.0±12.6 ^c^	5.6±4.7 ^bc^	4.1±3.5 ^bc^	27.8±15.5 ^c^	24.6±14.8 ^c^
Time 90	12.9±11.0 ^d^	18.9±14.3 ^d^	9.2±9.8 ^d^	14.4±11.7 ^d^	3.7±3.0 ^c^	4.5±3.8 ^c^	19.0±13.1 ^d^	22.1±16.3 ^d^

RBS: Repeat Breeder Syndrome. abcd: different superscripts indicate statistically significant differences within the same column and variable group (RBS and Pregnancy).

## Discussion

According to our results, there are no statistically significant differences between healthy and RB cows regarding the concentration of IgG and IgA in cervical mucus and sperm motility, which might indicate that the presence of immunoglobulins in cervical mucus is not a common cause of RB syndrome. Similarly, no differences were found between pregnant and non-pregnant cows concerning these two factors. On the other hand, as expected, time had a significant effect on sperm motility.

Previous studies reported that cows with high levels of anti-sperm antibodies (ASA) in cervical mucus presented reduced sperm motility compared to cows with low levels (Lazarevic et al., 2013). Similarly, Jarora et al. (2016) concluded that the presence of high ASA titre in cervical mucus may impair or delay fertility. However, it should be noted that there are noticeable differences between the design and the methodology of our study and the studies abovementioned. First, we aimed to determine if the concentration of immunoglobulins in cervical mucus varied significantly between healthy and RB cows, and if it might affect sperm motility, whereas other studies did not consider the RB factor. In the same way, the assays to determine the concentration of immunoglobulins differed, which may lead to differences in the limit concentration of immunoglobulins established as cut-off point to separate cows into groups. Additionally, our results showed that very few cows had high concentrations of immunoglobulins in cervical mucus (2/47 for IgG and 10/37 for IgA), which might impair the power of the statistical analysis.

In terms of cellularity, PMN count significantly differed in both RBS and pregnant groups, which might be explained by the fact that an adequate endometrial environment is fundamental for the establishment of pregnancy and survival of the embryo. This is in accordance with the consideration of subclinical endometritis as a possible cause of RB syndrome, as PMN infiltration in the endometrium has been previously reported in RB cows (Perez-Marin et al., 2012; Salasel et al., 2010).

## Conclusion

Although it has been proven that the concentration of immunoglobulins in cervical mucus affects sperm motility, we found no difference regarding this factor between cows with and without RB syndrome, which may indicate that a local immune response IgG/IgA-mediated against spermatozoa is not one of the common causes of this issue. However, our results may lack significancy due to the low number of cows and the consideration of a low cut-off point between groups. Therefore, further investigation is required to shed light on this matter.

## Data Availability

Research data is only available upon request.
